# Demographic, Clinical Features and Outcome Determinants of Thoracic Trauma in Sri Lanka: A Multicentre Prospective Cohort Study

**DOI:** 10.1155/2020/1219439

**Published:** 2020-06-19

**Authors:** Yasith Mathangasinghe, Iddagoda Hewage Don Saman Pradeep, Dhammike Rasnayake

**Affiliations:** ^1^Department of Anatomy, Faculty of Medicine, University of Colombo, Colombo, Sri Lanka; ^2^National Hospital for Respiratory Diseases, Welisara, Sri Lanka

## Abstract

Prognostic determinants in thoracic trauma are of major public health interest. We intended to describe patterns of thoracic trauma, demographic factors, clinical course, and predictors of outcome in selected tertiary care hospitals in Sri Lanka. A multicentre prospective cohort study was conducted in five leading teaching hospitals from June to September 2017. Patients with thoracic trauma were followed up during the hospital stay. A logistic regression analysis was conducted using in-hospital morbidity as the dichotomous outcome variable. One hundred seventy-one patients were included in the study yielding 1450 (median = 8.5) person-days of observation. Of them, 71.9% (*n* = 123) were males. The mean age was 45.8 ± 17.9 years. Majority (39.2%, *n* = 67) were recruited from the National Hospital of Sri Lanka. Automobile accidents were the commonest (62.6%, *n* = 107), followed by falls (26.9%, *n* = 46), assaults (8.8%, *n* = 15), and animal attacks (1.8%, *n* = 3). The ratio of blunt to penetrating trauma was 5.6 : 1. Injury patterns were rib fractures (80.7%, *n* = 138), haemothorax (44.4%, *n* = 76), pneumothorax (44.4%, *n* = 76), lung contusion (22.8%, *n* = 39), flail segment (15.8%, *n* = 27), tracheobronchial trauma (7.0%, *n* = 12), diaphragmatic injury (2.3%, *n* = 4), vascular injury (2.3%, *n* = 4), cardiac contusions (1.1%, *n* = 2), and oesophageal injury (0.6%, *n* = 1). Ninety nine (57.9%) had extrathoracic injuries. Majority (63.2%, *n* = 108) underwent operative management including intercostal tube insertion (60.8%, *n* = 104), wound exploration (6.4%, *n* = 11), thoracotomy (4.1%, *n* = 7), rib reconstruction (4.1%, *n* = 7), and video-assisted thoracoscopic surgery (2.9%, *n* = 5). Pneumonia (10.5%, *n* = 8), bronchopleural fistulae (2.3%, *n* = 4), tracheaoesophageal fistulae (1.8%, *n* = 3), empyema (1.2%, *n* = 2), and myocardial infarction (1.2%, *n* = 2) were the commonest postoperative complications. The mean hospital stay was 15.6 ± 18.0 days. The in-hospital mortality was 11 (6.4%). The binary logistic regression analysis with five predictors (age, gender, mechanism of injury (automobile/fall/assault), type of trauma (blunt/penetrating), and the presence of extrathoracic injuries) was statistically significant to predict in-hospital morbidity (*X*^2^ (6, *n* = 168) = 13.1; *p*=0.041), explaining between 7.5% (Cox and Snell *R*^2^) and 14.5% (Nagelkerke *R*^2^) of variance. The automobile accidents (OR: 2.3, 95% CI = 0.2–26.2) and being males (OR: 2.3, 95% CI = 0.6–9.0) were the strongest predictors of morbidity.

## 1. Introduction

Thoracic injuries are caused by 14% and 12% of the cases of blunt and penetrating trauma, respectively [1]. Moreover, 50% of the cases of polytrauma are associated with major thoracic injuries [2, 3], contributing to a significant morbidity and mortality worldwide [4–7]. The incidence of sepsis and multiorgan failure following thoracic trauma has gradually declined over the last 10 years [3]. Despite recent advancements in diagnostic and therapeutic trauma care, the mortality of severe thoracic trauma remains unchanged [3], which may account for up to 25% of the mortality in patients with polytrauma [8].

Regardless of the devastating complications associated with thoracic trauma, timely diagnosis and proper management can significantly reduce morbidity and mortality [9, 10]. Accurate diagnosis of thoracic trauma depends on a high degree of suspicion and pattern recognition [11]. Thus, knowledge on unique demographic patterns of presentation of thoracic trauma is invaluable in making an accurate diagnosis. Moreover, prediction of complications based on the presenting injury patterns may have a significant effect on the secondary prevention of complications [12, 13].

There are major differences of the reported demographic patterns, associated injuries, and complications of thoracic trauma in different regions of the world [14–16]. However, these data are sparse in the developing countries such as Sri Lanka, probably due to the unavailability of a national electronic trauma registry system. Therefore, the objective of this study was to describe morbidity and mortality of thoracic trauma and the determinants of in-hospital morbidity following thoracic trauma in five selected tertiary care hospitals in Sri Lanka.

## 2. Materials and Methods

The study was approved by the Ethics Review Committee, Faculty of Medicine, University of Kelaniya (EC/P/160/06/2017), and the protocol conformed to the guidelines set out by the Declaration of Helsinki [17].

A prospective cohort study was conducted in five leading teaching hospitals in Sri Lanka from June to September 2017. All the patients presenting with chest trauma to these hospitals during the study period were enrolled in the study. Patients who were transferred between the selected hospitals were excluded to prevent duplication of the results. The patients who died in the resuscitation room before obtaining computed tomography (CT) or X-ray were also excluded because of the inability to diagnose chest trauma accurately. On admission to the accident and emergency departments, patients and bystanders were interviewed to collect data. Eyewitness accounts for those with retrograde amnesia were also collected. Medical records of patients who were transferred from regional hospitals were also referred to obtain data. All patients were assessed by trauma surgeons to decide on the management. Patients were followed up daily during their hospital stay. The total hospital stay in days was presented to the nearest integer. The hospital stay was considered zero if the patients were managed in the outpatient departments or discharged within 12 hours of admission.

The dichotomous outcome variable was the development of major complications during hospital stay (hereafter referred to as “in-hospital morbidity”). Five predictors were selected: age, gender, mechanism of injury (automobile/fall/assault), type of trauma (blunt or penetrating), and the presence of extrathoracic injuries. A binary logistic regression model was fitted to examine if the independent variables effectively predicted the outcome. Animal attacks were excluded casewise from the model since there were only three such incidents. All the analyses were conducted at a priori alpha of 0.05.

## 3. Results and Discussion

### 3.1. Participant Flow

Two hundred and three potentially eligible patients were identified. Of them, eight had amnesia with no bystanders leaving 195 eligible for the study. Fifteen patients declined to participate in the study, thus 180 patients were interviewed. Nine patients were lost to follow-up due to transferring back to local hospitals after initial management (*n* = 7), leaving against medical advice (*n* = 1) and withdrawing from the study (*n* = 1). Hence, we considered 171 patients in the final analysis yielding 1450 (median = 8.5) person-days of observation.

### 3.2. Sociodemographic Features

The majority of the study population (71.9%, *n* = 123) was comprised of males. The mean age was 45.8 years (SD = 17.9). A major proportion of the participants (39.2%, *n* = 67) were directly recruited from the National Hospital of Sri Lanka, followed by Teaching Hospital Anuradhapura (32.2%, *n* = 55) and Colombo North Teaching Hospital (18.7%, *n* = 32). Of them, 42 (24.6%) were direct admissions to these tertiary care hospitals while the rest were transferred from the other referral centres in the country. Only one patient was referred from the private sector.

### 3.3. Injury Patterns

**Figure 1:**
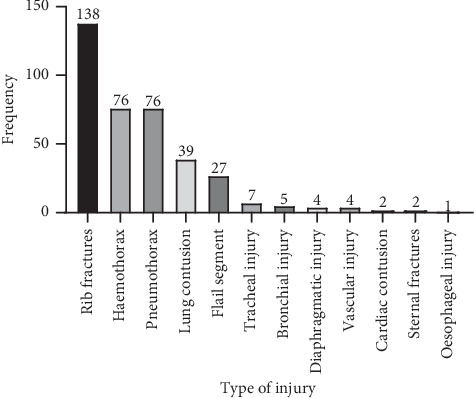
A bar chart illustrating the types of thoracic injuries diagnosed among the study participants (*n* = 171). Of those who had unilateral rib fractures, left hemithoracic fractures (*n* = 73) outnumbered right hemithoracic fractures (*n* = 49). Seventeen patients had bilateral rib fractures. All the tracheal injuries (*n* = 7) were located at the carinal level while all the bronchial injuries (*n* = 5) were lateralized to the left side. Vascular injuries were comprised of two subclavian arterial and two brachiocephalic arterial injuries. One patient had a mid-oesophageal level injury.

Automobile accidents were the commonest (62.6%, *n* = 107) aetiology for thoracic trauma followed by falls (26.9%, *n* = 46) and assaults (8.8%, *n* = 15). Three (1.8%) elephant attacks were reported from the North Central Province. The ratio of blunt trauma to penetrating trauma was 5.6 : 1. Rib fractures (80.7%, *n* = 138), haemothorax (44.4%, *n* = 76), and pneumothorax (44.4% *n* = 76) were the commonest types of injuries (Figure 1). The median of the ribs fractured was two (IQR = 1–5), with 26 having flail segments. Ninety nine (57.9%) had extrathoracic injuries. The commonest extrathoracic injury types were limb fractures (*n* = 54), head injury (*n* = 50), abdominal injuries (*n* = 25), and pelvic fractures (*n* = 19).

### 3.4. Management

The majority (63.2%, *n* = 108) underwent operative management including intercostal tube insertion (60.8%, *n* = 104), wound exploration (6.4%, *n* = 11), thoracotomy (4.1%, *n* = 7), rib reconstruction (4.1%, *n* = 7), and video-assisted thoracoscopic surgery (VATS) (2.9%, *n* = 5). Thirty five (20.5%) were intubated on admission and 46 (26.9%) eventually received mechanical ventilation. Paracetamol (83.0%, *n* = 142) was the commonest mode of analgesia. Paracetamol was given as the only analgesic in 12 (7.0%) cases, while it was combined with opioids and nonsteroidal anti-inflammatory drugs (NSAIDs) in 130 (76.0%) and 114 (66.7%) cases, respectively. In all these instances, the routes of administration were oral, rectal, or intramuscular. Epidural (2.3%, *n* = 4), intrapleural (1.8%, *n* = 3), and paravertebral (0.6%, *n* = 1) analgesia were rarely utilized. Of those who received the abovementioned types of regional anaesthesia had a median of seven (IQR = 6–8) ribs fractured with six out of eight cases having flail segments.

**Figure 2:**
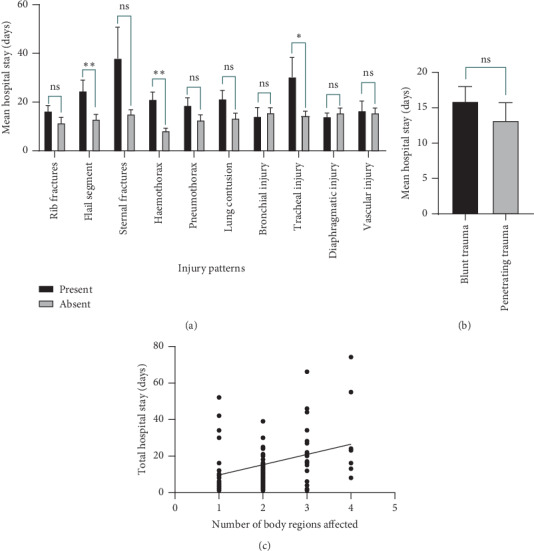
Factors associating with the length of the hospital stay. (a) A bar chart comparing the mean hospital stay in different types of injuries. The level of significance of the independent sample *t-*tests comparing the mean hospital stay depending on the presence or the absence of the individual injury type is denoted above each pair of columns. (b) A bar chart comparing the mean hospital stay in blunt and penetrating trauma. (c) A scatter plot illustrating a positive correlation between the length of the hospital stay and the number of body regions affected. Pearson's correlation coefficient *r* **=** 0.293, *p*=0.004. Note: the affected regions of the body were categorized as the head and neck, thorax, abdomen, pelvis, and extremities. Error bars indicate the standard error of the means (SEM). ns, not significant; ^*∗*^*p* < 0.05; ^*∗∗*^*p* < 0.01.

The mean hospital stay was 15.6 ± 18.0 days. The presence of a flail segment, haemothorax, or a tracheal injury was associated with prolonged hospital stay (Figure 2(a)). However, the duration of the hospital stay did not vary according to the pattern of injury (i.e., blunt versus penetrating trauma) (Figure 2(b)). A positive correlation was found between the number of body regions affected and the duration of hospital stay (*r* = 0.293, *p* = 0.004) (Figure 2(c)).

### 3.5. Complications

Pneumonia (10.5%, *n* = 18), bronchopleural fistulae (2.3%, *n* = 4), tracheaoesophageal fistulae (1.8%, *n* = 3), empyema (1.2%, *n* = 2), and myocardial infarction (1.2%, *n* = 2) were short-term postoperative complications detected during the period of hospital stay (see Supplementary Table 1 for a comparison of the complications between blunt and penetrating trauma). Pearson's chi square test with Yate's correction did not show significant associations between the presence of pulmonary complications and the pattern of injury (i.e., blunt versus penetrating trauma) (*X*^2^ (1, *n* = 171) = 0.599; *p*=0.44). Eleven patients (6.4%) died during the hospital stay. Of them, seven patients had combined haemopneumothoraces, lung contusions, and extrathoracic injuries.

### 3.6. Logistic Regression

Preliminary tests confirmed that there were no violations of assumptions of normality and multicollinearity. The full model of the binary logistic regression analysis containing five predictors (age, gender, mechanism of injury (automobile/fall/assault), type of trauma (blunt or penetrating), and the presence of extrathoracic injuries) was statistically significant to predict in-hospital morbidity (*X*^2^ (6, *n* = 168) = 13.1, *p*=0.041). The model as a whole explained between 7.5% (Cox and Snell *R*^2^) and 14.5% (Nagelkerke *R*^2^) of variance for in-hospital morbidity. Motor vehicle accidents (OR: 2.3, 95% CI = 0.2–26.2) and being males (OR: 2.3, 95% CI = 0.6–9.0) were the strongest predictors of morbidity. Moreover, having extrathoracic injuries, penetrating injuries, and young age increased the likelihood of developing in-hospital morbidity (Supplementary Table 2).

## 4. Discussion

Sri Lanka is a country in South Asian region which is house for over 20 million people. Being a developing country, there is only one specialized centre for thoracic surgery, with several tertiary care hospitals having facilities to conduct basic thoracic surgical procedures. In this multicentre prospective cohort study, we recruited patients from five leading tertiary care hospitals with thoracic surgical facilities inclusive of the specialized centre for thoracic surgery in the country. These hospitals accept patients from all over the country, and thus the participants in this study represented different territories of Sri Lanka. We found that most of the patients affected with thoracic trauma were males (71.9%) and the majority were automobile accidents (62.6%), while the mean age of the victims was 45.8 years, despite a slight female predominance in the Sri Lankan population [18]. Nevertheless, the demographics of our study is in line with a recent meta-analysis of blunt thoracic trauma patients which concluded that the mean age of presentation was 45.7 years with a male preponderance (74%) [19]. More than two-thirds of the thoracic trauma are due to automobile accidents in both the Western [20, 21] as well as regional countries [22], whereas this proportion in our study was slightly less than two-thirds. Nonetheless, encountering automobile accidents and male gender were associated with poor outcomes in this study.

The commonest injury pattern we observed was rib fractures (80.7% of the patients). A retrospective study conducted in a rural region of India reported that only 28.8% of hospitalized thoracic trauma patients had rib fractures [22]. Similarly, a retrospective cohort study based on a national trauma registry in Germany reported that up to 38.6% of patients with thoracic trauma had rib fractures or unilateral flail chest [21]. The possible reason for the comparatively higher number of rib fractures reported in our study is that the patients were recruited from tertiary care hospitals, where patients with minor thoracic trauma may have been treated in the regional primary care hospitals.

The sequalae of thoracic trauma is diverse, with a significant morbidity associated with secondary complications [3]. Blunt injuries are reported to be associated with poor clinical outcomes compared to penetrating injuries [3, 23, 24]. Intriguingly, however, we observed increased morbidity in patients with penetrating trauma (Supplementary Table 2). Nonetheless, this predictor had only a weak association with morbidity in our logistic regression model. In our series, 21 (12.3%) patients developed secondary complications during the hospital stay. Even simple rib fractures can cause significant complications due to parenchymal lung injury and hypostatic pneumonia caused by pain [1]. Having pulmonary injuries is an independent risk factor for the development of multiorgan dysfunction syndrome in trauma patients [25]. Therefore, a high degree of suspicion is necessary to diagnose pulmonary complications associated with thoracic trauma.

Patients presenting with blunt thoracic trauma are assessed for their pain, and analgesics are administered according to the analgesic ladder starting with paracetamol, opioids, and NSAIDs. Patient control analgesia, infusions of local anaesthetics, paravertebral blocks, or epidural analgesia is considered subsequently if the pain does not resolve with the initial medications. Eastern Association for the Surgery of Trauma and Trauma Anaesthesiology Society have developed guidelines on blunt thoracic trauma management in 2016 based on five meta-analyses [26]. They have recommended epidural and multimodal anaesthesia over intrapleural and intercostal analgesia in thoracic trauma. Nonetheless, a systematic review and a meta-analysis concluded that continuous epidural analgesia has no superior clinical outcomes when used to treat patients with traumatic rib fractures [27]. Intriguingly, epidural analgesia was used only in four patients (2.3%) according to the present study. Moreover, we observed that pain management was largely restricted to oral, rectal, and intramuscular modes of analgesia with minimum use of other modalities in our study settings. The reasons for the infrequent administration of other modalities of analgesia in Sri Lankan hospitals for thoracic trauma must be explored and necessary steps should be taken to overcome this shortcoming in the future.

## 5. Limitations

The main limitation of this study is that the patients were followed up only during the hospital stay. Therefore, intermediate- and long-term morbidity and mortality data were not available. Since this study was conducted only in the tertiary care hospitals, patients with less severe injuries who would have been managed in the primary care settings are underrepresented. Contrarily, since patients with severe polytrauma who died in the emergency resuscitation room were not assessed in this study, it may have underreported severe injury patterns. We did not use an indicator to assess the global severity of trauma such as Injury Severity Score in the predictive model since the majority of trauma surgical centres in the developing countries do not regularly use them in the patient management [28]. Furthermore, we did not statistically analyse the predictors of mortality since the number of patients who died in this cohort was 12 (6.4%).

Sri Lankan government provides free healthcare to Sri Lankan citizens. However, the government sector is unable to supply rib fixation devices free of charge; hence, there is a considerable delay in rib fixation. In fact, many patients are unable to afford these devices, hence managed conservatively. This limitation might have resulted in prolonged hospital stay of the patients in our cohort.

## 6. Conclusions

Rib fractures, haemothorax, and pneumothorax were the commonest patterns reported from the tertiary care hospitals studied. Motor vehicle accidents and male gender were associated with a high in-hospital morbidity.

## Data Availability

The data used to support the findings of this study are available from the corresponding author upon request.

## Supplementary Materials

Supplementary Materials Supplementary Table 1: short-term postoperative complications associated with the type of injury. Supplementary Table 2: logistic regression analysis predicting the likelihood of developing hospital morbidity.
