# Supplementary material for: Demographic, Clinical Features and Outcome Determinants of Thoracic Trauma in Sri Lanka: A Multicentre Prospective Cohort Study
Source: Can Respir J. 2020 Jun 19;2020:1219439. doi: 10.1155/2020/1219439 (PMC7322612; doi:10.1155/2020/1219439)
Supplement: Supplementary Materials — Supplementary Table 1: short-term postoperative complications associated with the type of injury. Supplementary Table 2: logistic regression analysis predicting the likelihood of developing hospital morbidity. [file 1219439.f1.docx]

**Supplementary Table 1**: Short-term postoperative complications associated with the type of injury

| Complication | Frequency based on the type of injury | | Total |
| --- | --- | --- | --- |
|  |  |  |  |
|  | **Blunt Trauma** | **Penetrating Trauma** |  |
| Pneumonia | 17 | 1 | 18 |
| Bronchopleural fistulae | 3 | 1 | 4 |
| Trachea-oesophageal fistulae | 3 | 0 | 3 |
| Empyema | 2 | 0 | 2 |
| Myocardial infarction | 2 | 0 | 2 |

**Supplementary Table 2**: Logistic regression analysis predicting the likelihood of developing in-hospital morbidity

|  | B | S.E. | Wald | df | *p* | Odds Ratio | 95% CI for Odds Ratio | |
| --- | --- | --- | --- | --- | --- | --- | --- | --- |
|  |  |  |  |  |  |  | Lower | Upper |
| Sex |  |  |  |  |  |  |  |  |
| Male | .836 | .694 | 1.452 | 1 | .228 | 2.307 | .592 | 8.981 |
| Age (in years) | -.003 | .015 | .030 | 1 | .863 | .997 | .969 | 1.027 |
| Mechanism |  |  |  |  |  |  |  |  |
| Automobile | .820 | 1.249 | .431 | 1 | .512 | 2.270 | .196 | 26.253 |
| Fall | -1.131 | 1.561 | .525 | 1 | .469 | .323 | .015 | 6.877 |
| Type of trauma |  |  |  |  |  |  |  |  |
| Blunt injuries | -.174 | .956 | .033 | 1 | .856 | .841 | .129 | 5.469 |
| Extrathoracic injuries |  |  |  |  |  |  |  |  |
| Not present | -.954 | .611 | 2.436 | 1 | .119 | .385 | .116 | 1.276 |
| Constant | -2.571 | 1.339 | 3.685 | 1 | .055 | .076 |  |  |

Abbreviations: B – unstandardized beta; CI – confidence interval; df – degrees of freedom; *p* – significance; S.E. – standard error;
